# Supplementary figures and images for: TolRad, a model for predicting radiation tolerance using Pfam annotations, identifies novel radiosensitive bacterial species from reference genomes and MAGs
Source: Microbiol Spectr. 2024 Sep 5;12(10):e03838-23. doi: 10.1128/spectrum.03838-23 (PMC11466087; doi:10.1128/spectrum.03838-23)

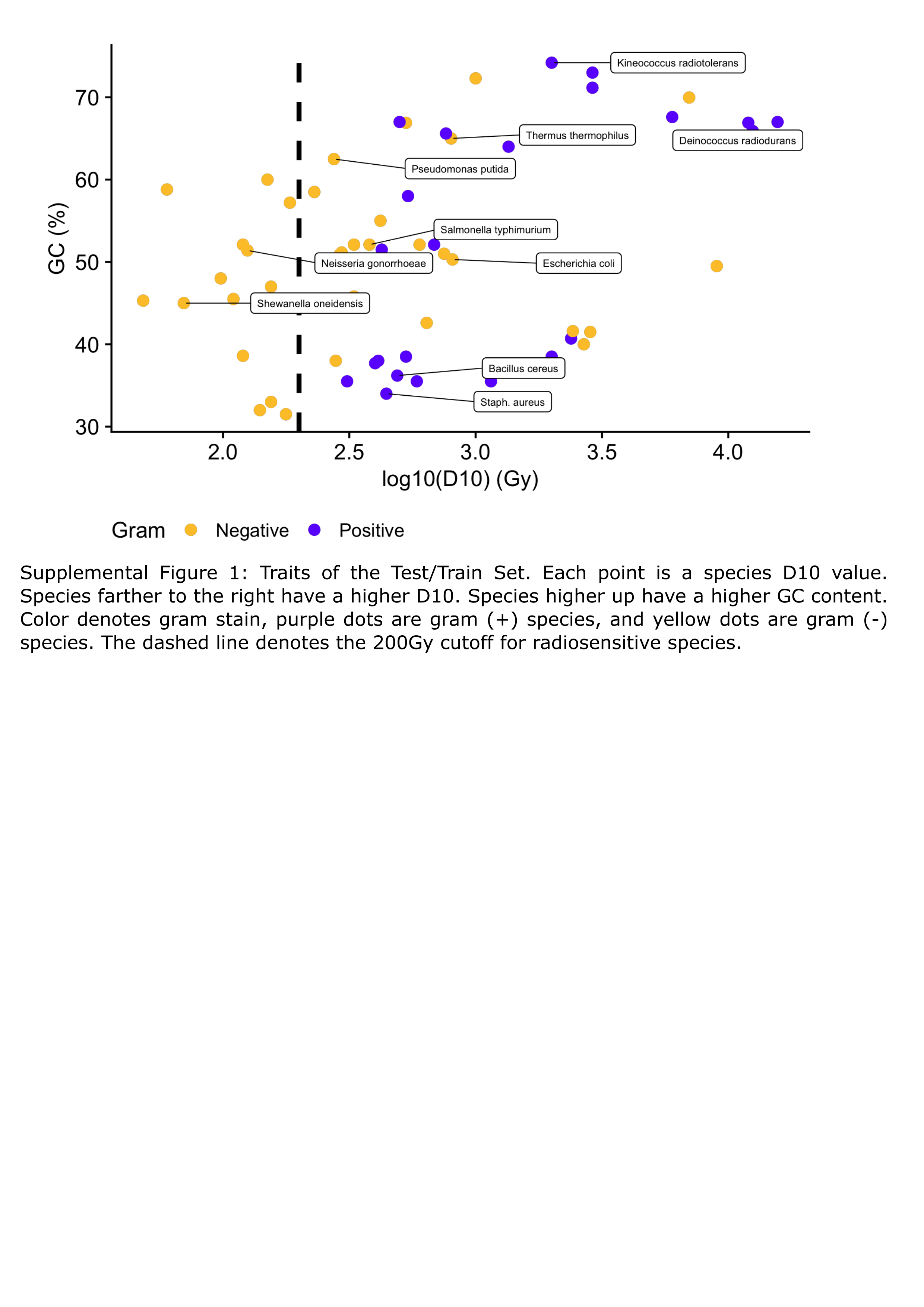

Supplement: Supplemental Figure 1 — Traits of the Test/Train set. [file spectrum.03838-23-s0001.tiff]

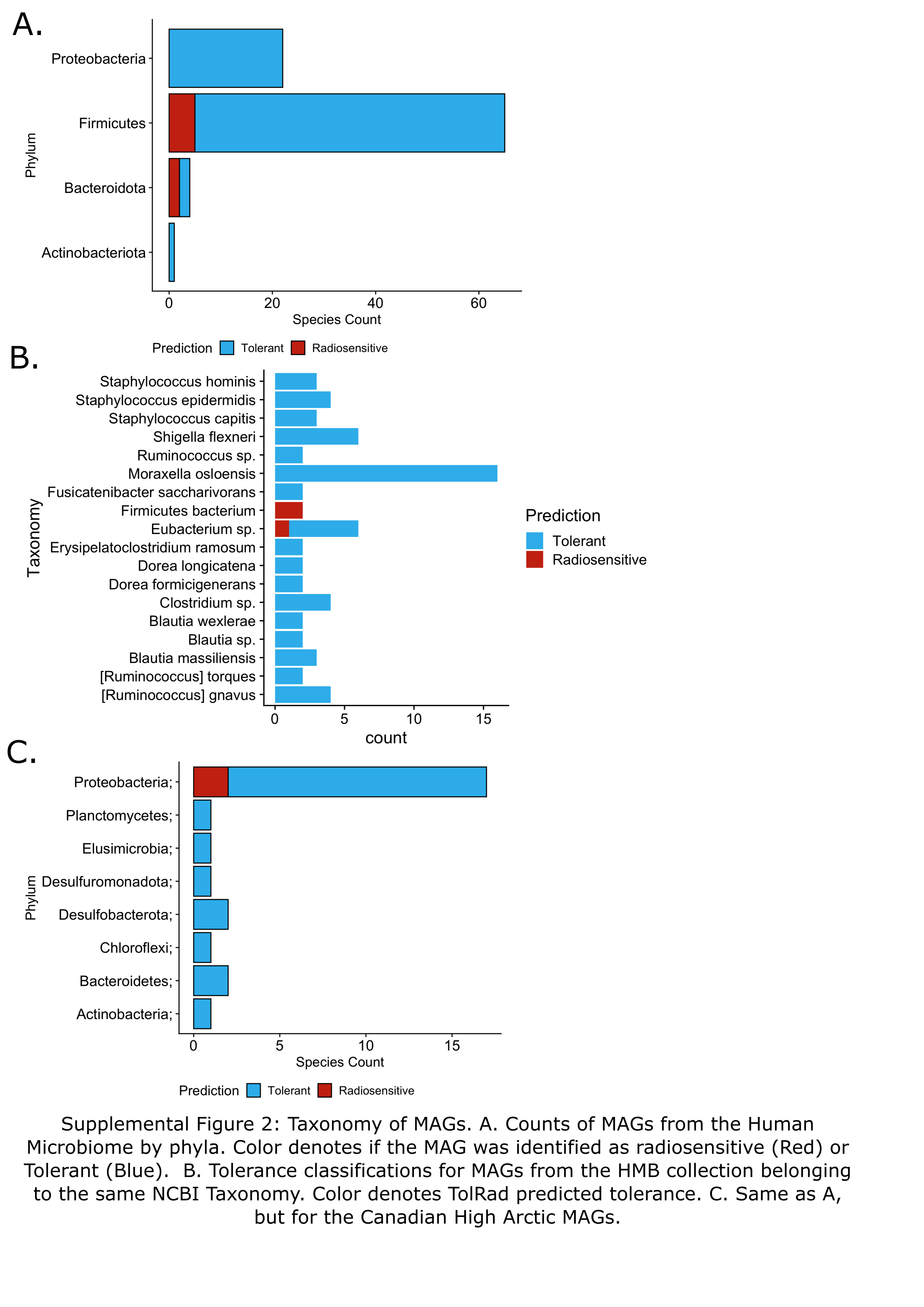

Supplement: Supplemental Figure 2 — Taxonomy of MAGS. [file spectrum.03838-23-s0002.tiff]
